# Emotional Intelligence and Well-Being of Special Education Teachers in China: The Mediating Role of Work-Engagement

**DOI:** 10.3389/fpsyg.2021.696561

**Published:** 2021-08-30

**Authors:** Wangqian Fu, Chonggao Wang, Wenjun Tang, Sha Lu, Yan Wang

**Affiliations:** ^1^School of Special Education, Faculty of Education, Beijing Normal University, Beijing, China; ^2^Changsha Institute of Educational Science, Changsha, China; ^3^Beijing Institute of Educational Science, Beijing, China

**Keywords:** well-being, special education teachers, work engagement, emotional intelligence, China

## Abstract

The well-being of special education teachers is key to their mental health and also influences the development of their students. This study aimed to investigate the psychological mechanism of the well-being of special education teachers in China, where they are maximum in number. We explored the role of emotional intelligence (EI) and work engagement on the well-being of teachers. A total of 496 Chinese special education teachers participated in the current study. Results suggested that (1) the EI and work engagement were positively associated with the well-being of special education teachers; and that (2) work engagement played a mediating role on EI and well-being of special education teachers. To promote the well-being of special education teachers, suggestions for policymakers and schools are discussed.

## Instruction

Teaching is a highly emotional profession associated with high levels of stress that may be the cause of job dissatisfaction, psychological disorders, and reduced well-being (Chang, 2009; Brackett et al., 2010; Keller et al., 2014). Teachers frequently have to cope with stressful situations that can affect their well-being at work. Well-being is an important factor to reduce the intention of teachers to leave the career (Li, 2013), which affects the level of mental health, job performance, and professional identity of special education teachers (Ma and Deng, 2019; Wang, 2017), and therefore it has become an important reference for measuring the stability of the teaching team to a certain extent and has become an important part of professional development of teachers (Holmes, 2005; Lam, 2019). Besides, the level of well-being of the teachers within schools has been found to be linked to pupil performance (Briner and Dewberry, 2007), which implies that improving well-being among employees has benefits not only for the employees themselves, but also for the success of the organization (Giorgi et al., 2011).

Special education teachers face greater professional pressure in teaching (Major, 2012; Shyman, 2011), especially for those in China, working in the special education schools that have a separate curricular standard, evaluation, administration, and so on (Fu et al., 2019). Although influenced by the philosophy of inclusive education from the western countries, special education schools are still the main educational places for children with disabilities in China. There were 2,192 special education schools in China in 2019, where 28.87% of students with disabilities were studying and 62,400 special education teachers were teaching (The Ministry of Education of China, 2020). An empirical study shows that Chinese special education teachers experience lower professional well-being than the regular school teachers (Zhao and Huang, 2015). It has been found that 25.63% of the special education teachers in China are having psychological problems, which is not only higher than that for general teachers in primary and secondary schools, but also much higher than that for the normal population of 5% (Tian et al., 2009). Furthermore, the psychological problems of special education teachers contribute to their higher job burnout and lower well-being (Zhang and Wei, 2014). Aiming to improve the quality of special education, the Chinese government has attached an increasing importance to the development of special education teachers and has issued a series of important policy documents to improve the positive professional experience of teachers, and ultimately benefited students with special needs in the last decade. Therefore, exploring the well-being of special education teachers in China with the largest special education system and analyzing the influence paths will help the government and the society to take measures needed to improve the well-being of special education teachers and contribute to the policy.

### The Impact of Emotional Intelligence on the Well-Being of Teachers

Emotional intelligence (EI) is the ability of an individual to identify her/his own emotions and emotions of others, and thus to form a favorable relationship with others (Salovey and Mayer, 1990). Based on the Conservation of Resources (COR) Theory (Hobfoll, 1989, 2001), EI can be regarded as an internal (emotional) resource, which could effectively explain the asymmetric relationship between EI and its outcome variables (Liao and Yan, 2014). Teachers experience a wide range of positive and negative emotions when teaching students (Brackett and Katulak, 2006). The nature of their job requires dealing with their own emotions as well as that of students, parents, colleagues, and administrators. EI has been seen as an important factor within the teaching profession and some relevant evidence have shown that the EI trait is likely to be one of the personality characteristics that possibly affect the experience of burnout and job satisfaction in teachers (e.g., Chan, 2004, 2006), while some other studies in the literature have shown that EI is a major resource for teaching professionals due to its associations with burnout and psychological distress (Mérida-López and Extremera, 2017; Mérida-López et al., 2017). In fact, EI is increasingly playing a crucial role in the occupational health models of teachers (Taris et al., 2017), which is related to higher satisfaction with life (Landa et al., 2006), increased teaching satisfaction (Yin et al., 2013), and more positive attitudes toward teaching (Mérida-López and Extremera, 2017).

Every day, special education teachers at work must apply their EI to interpret situational cues and decide how best to feel and respond. EI can help to change the attitudes and behaviors of employees at work, thereby increasing job satisfaction (Lee and Ok, 2012). Special education teachers with high-perceived EI are likely to experience less burnout, and thus greatly improve their job-related well-being (Li, 2016).

### The Relationship Between Work Engagement and the Well-Being of Teachers

Work engagement is a positive affective–emotional state and sense of accomplishment that includes three dimensions: vigor, dedication, and absorption (Schaufeli and Bakker, 2010). Work engagement has been frequently studied through the job demands–resources model developed by Bakker and Demerouti (2007), and becomes essential when studying subjective well-being at work (Bakker and Oerlemans, 2011). There are many advantages to people who have high level of work engagement. Engaged teachers are believed to be less prone to burnout and associated health problems (Hakanen et al., 2006), thus linking level of engagement inversely with teacher attrition. Simply put, engaged teachers are less likely to quit the profession or require expensive support for health-related problems. Developing a better understanding of the engagement of teachers at work may lead to insight into ways to enhance the well-being of teachers and to build the effectiveness of teachers in the classroom.

Previous studies indicated that well-being and work engagement are positively associated, and they impacted each other (Shimazu and Schaufeli, 2009; Shimazu et al., 2012; Upadyaya and Salmela-Aro, 2013). Special education teachers face high stress due to the limitations of students in the development of intellectual, physical, social, and emotional aspects resulting in their slow progress, while parents demanded quick results (Lestari and Sawitri, 2017). Besides the influence of students, special education teachers in China who lack practical training and professional skills, working in schools with high student–faculty ratios and insufficient facilities (Lai et al., 2016), are in severe stress. Heavy workload can make special education teachers unenergetic and not enjoy their job, which indicates a low work engagement (Chen et al., 2010). It has been demonstrated that there is a positive relationship between work engagement and well-being of general teachers (Klassen et al., 2012), while a few scholars have explored this key topic regarding special education teachers.

### The Impact of Work Engagement Between EI and Well-Being

Consistent with the JD–R model, social and personal resources such as EI would moderate the associations between job demands and organizational outcomes. Accordingly, EI as a personal resource might energize employees, encourage their persistence, and make them focus on their efforts. In other words, these emotional resources might foster engagement in terms of vigor (energy), dedication (persistence), and absorption (focus) (Demerouti et al., 2001; Extremera et al., 2012; Bakker et al., 2014). Positive links between EI and teacher engagement have been consistently reported (Pena et al., 2012). EI is considered as an antecedent of work engagement (Bakker et al., 2014). The EI of special education teachers has a significant positive predictive effectiveness for work engagement (Li, 2016). Besides, research finds that positive EI can enhance well-being by increasing the work engagement of teachers (Brunetto et al., 2012; Bermejo-Toro et al., 2016).

Despite the progress in recent research with regard to measuring the well-being of special education teachers and exploring the relationship among the EI, work engagement, and well-being of special education teachers, some literature gaps still exist. First, the investigation of well-being of special education teachers in developing countries is limited. China, with more than 58,000 special education teachers, is a natural laboratory for conducting preliminary research on the well-being of special education teachers, which may contribute to the international research on the topic. Second, although there is some literature on the relationship between the EI and well-being with regard to the special education teachers, there have been only a few attempts to examine the interrelationship among the EI, work engagement, and well-being of special education teachers.

To address these gaps, in the present study we explored the level of well-being of Chinese special education teachers and the relationship of EI, work engagement, and well-being, especially the effect of work engagement between EI and well-being. Based on the conceptual framework and the empirical evidence collected in the literature review, three hypotheses are proposed to be tested with our survey data.

**H1:** EI has a positive effect on the well-being of Chinese special education teachers.

**H2:** Work engagement has a positive impact on the well-being of special education teachers in China.

**H3:** Work engagement plays a mediating role on the relationship between EI of Chinese special education teachers and their well-being.

## Materials and Methods

### Participants and Procedure

Data were collected online though questionnaires from 67 special education schools in five provinces in Mainland China. Participants were eliminated according to the criteria that the same number of questionnaires were more than 90%. A total of 496 valid questionnaires were collected, with a recovery rate of 97.64%.

### Measures

#### Emotional Intelligence Scale

This study adopted the EI scale compiled by Wong and Law (2002). The scale consists of 12 items (e.g., “I have a good understanding of my emotions.” and “I really understand how I feel.”) on a 5-point Likert-type scale, from “totally inconsistent” to “very consistent.” The higher the score, the higher the EI.

#### Work Engagement Scale

The UWES Work Engagement Scale developed by Schaufeli et al. (2002a, b), was used in this study. The Chinese version was revised by Zhang and Gan (2005). It contains tripartite: vitality (six items), dedication (five items), and focus (six items). It requires respondents to use a 5-point Likert scale to evaluate the extent to which they experience these feelings, from “totally inconsistent” to “very consistent.” The higher the score, the higher the level of work input.

#### General Well-Being Scale

The scale is a formulaic test tool developed by the National Center for Health Statistics to evaluate the statements of happiness by the subjects. The scale was revised by Duan (1996). The revised scale has 18 items, including six factors: worry about health, energy, satisfaction and interest in life, melancholy or happy mood, control of emotion and behavior, relaxation, and tension. It requires respondents to use a 5-point Likert scale to evaluate the extent to which they experience these feelings, from “totally inconsistent” to “very consistent.” There are nine questions in the reverse scorecard. The higher the score, the higher the general well-being.

### Data Analysis

In the analysis, the valid sample size is 496. EI, work engagement, and overall well-being were the observed variables. The standard score of each dimension was taken as the value of each observation variable. To test whether work engagement plays a mediating role in EI and general well-being, the mediation model was applied using the PROCESS SPSS computing tool.

## Results

### Descriptive Statistics

The sample consisted of 78.23% women and 21.77% men. There were 22.18% of the sample of low teaching experience—up to 3 years of teaching, 12.5% of the participants with 3–5 years of teaching experience, and 16.33% of the respondents having 6–10 years of teaching experience, and the rest (48.99%) have the high teaching experience—more than 10 years of teaching.

The reliability of the questionnaire is calculated using the Cronbach alpha coefficient. Confirmatory factor analysis was conducted to test item factor loading. The reliability and factor-loading range of each questionnaire are shown in Table 1.

**TABLE 1:** Questionnaire reliability and factor loading.

**Variable**	**Cronbach’s alpha**	**Factor loading**
Emotional intelligence	0.945	0.694–0.815
Work engagement	0.956	0.634–0.848
General well-being	0.858	0.369–0.796

### Correlations Among EI, Work Engagement, and General Well-Being

The study also investigated the relationship among all variables. To this aim, Kendall’s tau-b correlation coefficient measure was conducted on EI, work engagement, and general well-being responses. Variables with descriptive and correlation statistics are presented in Table 2.

**TABLE 2:** Variables descriptive and correlative statistics.

**Variable**	***M* ± SD**	**1**	**2**	**3**
Emotional intelligence	45.81 ± 7.13	1		
Work engagement	62.92 ± 11.68	0.52**	1	
General well-being	75.57 ± 13.54	0.30**	0.31**	1

**p < 0.01.

As Table 2 indicates, there were positive correlation coefficients between EI, work engagement, and general well-being. EI was significantly associated with work engagement (*r* = 0.52, *p* < 0.01) and general well-being (*r* = 0.30, *p* < 0.01). Meanwhile, work engagement was significantly associated with general well-being (*r* = 0.31, *p* < 0.01). Therefore, it can be inferred that the higher the EI or the work engagement, the higher the general well-being.

### The Mediation Effect of Work Engagement

Confirmatory factor analysis was conducted to test the latent structure of each scale. As show in Table 3, all single-factor models achieved the acceptable model fit.

**TABLE 3:** Confirmatory factor analysis of each scale.

**CFA**	**CFI**	**TLI**	**RMSE**	**SRMR**
Work engagement	0.92	0.91	0.09	0.05
General well-being	0.83	0.79	0.09	0.06
Emotional intelligence	Just identified			

**FIGURE 1:**
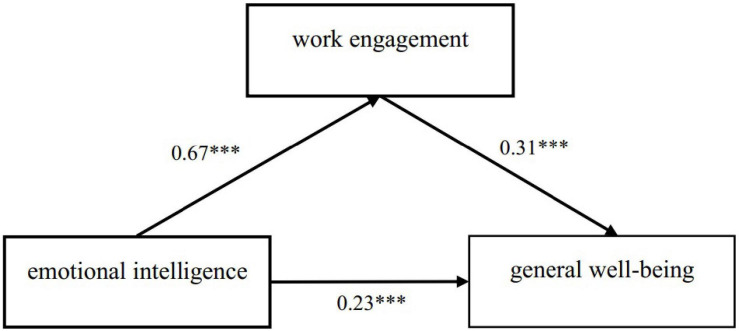
Work engagement as a mediator between emotional intelligence and general well-being. ***p < 0.001, **p < 0.01, *p < 0.05.

Then Structural Equation Model was conducted to explore the role of EI and work engagement on general well-being. The model shows that work engagement mediates the influence of EI on general well-being (see Figure 1).

The standard score of each dimension is taken as the value of each observation variable. We tested whether work engagement plays a mediating role in EI and general well-being (see Table 4). The result showed that the model had a direct role on the influence of EI on general well-being, and an indirect path from EI to work engagement, and then from work engagement to general well-being. The total effect = 0.44, the direct effect = 0.23, the indirect effect = 0.21, and 95% percentile bootstrap confidence intervals = (0.12, 0.29); this interval does not include 0, indicating that the mediating effect is significant.

**TABLE 4:** Mediation analyses on emotional intelligence and general well-being (5000 bootstraps).

**Independent variable (IV)**	**Dependent variable (DV)**	**Mediator (M)**	**Effect of**		**Effect of**		**Effect of**		**Direct**		**Indirect**		**Total**	
			**IV on M**		**M on DV**		**IV on DV**		**effect**		**effect**		**effect**	
			**Coefficient**	**SE**	**Coefficient**	**SE**	**Coefficient**	**SE**	**Coefficient**	**SE**	**Coefficient**	**95% CI**	**Coefficient**	**SE**
Emotional intelligence	General well-being	Work engagement	0.67***	0.03	0.31***	0.05	0.23***	0.05	0.23***	0.05	0.21***	0.12, 0.29	0.44***	0.04

***p < 0.001.

## Discussion

The study was carried out in a sample of Chinese special education teachers to explore the influencing factors of their well-being and understanding the roles of EI and work engagement.

Correlation analyses confirmed that there is a positive correlation between EI, work engagement, and general well-being. The positive association between EI and work engagement of special education teachers is consistent with previous studies by Lestari and Sawitri (2017) and Li (2016). Those studies about special education teachers indicated that high spirit and work engagement of teachers are directed by their high EI. For special education teachers reported high scores in EI, they perceived themselves as doing well in managing self-relevant information, in regulating optimism/mood, and in repairing emotions by themselves, and also as doing moderately well in managing emotions of others (Platsidou, 2010), who would control themselves at work better and experience less distress (Nikolaou and Tsaousis, 2002). A high-trait EI is important in facilities work engagement and promoting professionals’ well-being. People with EI have motivation and experience positive emotions by cleverly managing their emotions. The experience of positive emotion with resilience can help special education teachers to be more involved in their work. That it EI of special education teachers contribute to their work engagement. High work engagement is shown by persistent efforts of teachers in finding teaching methods for students, a sense of pride in their work, and passion to help the students. In addition, to teach students with basic instruction, students also need to be guided to develop their talents. Special education teachers of high EI desire to help children with special needs so they can be independent and also empathize with the children, where they understand the difficulties of the students. In addition, they can also face and deal with misbehavior of children that may be impolite, and avoid negative feelings. Thus, special education teachers with a high EI can transmit their confidence to work enthusiastically and bond with their work.

Second, we found that the EI of teachers has an affirmative effect on their well-being. That is, Chinese special education teachers with a high ability of emotion perception have a higher experience of subjective well-being. It is in line with and strengthens further the results obtained among teachers by Tan (2019) and Blasco-Belled et al. (2020). The studies showed that the ability to perceive, understand, and regulate one’s own emotions and that of others is necessary to develop in order to achieve better emotional and personal well-being. That is high levels of EI resulting in greater well-being (Fernández-Berrocal et al., 2017). For this reason, enhancing individual EI will promote social–emotion competence and well-being. EI may act to influence or moderate how individuals monitor and display their emotions. Especially mood repair is a positive predictor for well-being. Austin et al. (2008) suggested that individuals with high EI are less likely to express superficial emotions merely because it is expected of them, but instead they channel them appropriately and express them overtly. For the special education teachers, EI is a kind of emotion regulation benefit to their well-being. The ability to regulate or manage one’s emotions and to express them in a manner that suits specific situations is a kind of strategy to handle with their work stress and interpersonal adaptation. Teachers of that ability are likely to experience higher well-being. Chinese special education teachers with high EI can perceive some subtle emotions around well, and deal with the discomfort caused by negative emotions randomly. Special education highly demands teachers to cooperate with other stakeholders, including general education teachers, parents, school administrators, and others, which make the ability to understand others and reasoning accurately is critical. Besides, special education teachers may face various emotional distress by heavy work load and limited progress of students with disabilities (Fu et al., 2019), which requires them to have high skills to release their own negative emotions in a reasonable way to reduce their inner depression and improve their own happiness.

Pearson’s correlation revealed that there is a positive correlation between work engagement and subjective well-being of special education teachers. The positive correlation between work engagement and well-being is consistent with previous studies about special education by Fu et al. (2020). The higher the work engagement is, the higher the subjective well-being would be. When people are engaged in an activity whose task difficulty is equivalent to that of a skill, they should devote themselves to the activity and pay high attention to it, so as to achieve a kind of psychological experience when the activity and consciousness are fused, the sense of time disappears, and the state of selflessness is achieved. When an individual is in the process of work, the rich internal and external work resources can stimulate the positive working state of an individual and lead him to the psychological state of work engagement, which is conducive to the realization of work goals, and the individual can experience positive emotions and work satisfaction. The sense of mastery is a good experience for a happy life, which helps to improve the subjective well-being of individuals.

The regression analysis demonstrated that the levels of well-being were significantly predicted only by certain factors of work engagement. It is found that work engagement as an intermediary factor affects EI and well-being. That means the well-being of special education teachers can be influenced by work engagement through EI. Specifically, special teachers with a higher level of work engagement can make more frequent use of EI in their work, and maintain the satisfaction and happiness experience brought by mobilizing more psychological resources to manage and using their emotions. While special teachers with lower level of work engagement use less EI in their work, they experience less happiness due to less sense of gain from work involvement and feelings of efficacy in dealing with emotional problems.

### Practical Implication

First, take several measures to cultivate the EI ability of special education teachers. EI as a main protective factor of work engagement and well-being of special education teachers should be promoted. First, they have to master interpersonal communication skills to improve the ability of emotional perception and expression. The emotional expression of special education teachers not only has a great impact on their EI, but also has an important impact on students. Bad language habits will hinder the emotional cognition and experience of students, affect the interest of students in learning, and thus affect the teaching ability of teachers and classroom teaching effect. Therefore, special education teachers should reflect on the characteristics of their own emotional expression, appropriate use of action, expression vivid, accurate transmission of information to students, and effectively improve the ability of emotional control and use. Second, we should pay attention to empathy and improve the ability of emotional assessment and communication. Special education teachers have a high level of empathy, which is conducive to the positive emotional interaction between teachers and students, and deepens the emotional experience of each other, and promotes the smooth development of various educational and teaching works. Therefore, teachers should take the initiative to break the traditional concept of teacher–student hierarchy, establish a democratic and equal relationship between teachers and students, guide the emotional experience of students with personal emotional charm, at the same time, they should care about students, love students, learn to put themselves in the perspective of students to transpose thinking, understand students, patiently communicate and guide, nourish the heart of students with tolerance and understanding, and stimulate their enthusiasm and self-motivation, correct their deviation and misunderstanding. In addition, special education teachers should learn how to regulate and control emotions and enhance the ability of emotion management. Children with disabilities may have problems in action or communication, which need to be dealt with by special education teachers as a daily work. A teacher, who is naturally a role model, should be good at using reason to control emotions. Of course, improving the EI of special education teachers not only relies on the teachers themselves, but also the educational administrative departments and schools that should raise their awareness, attach importance to further study and training of special education teachers, actively carry out the psychological health education of teachers, and promote the professional development of special education teachers through internal and external forces.

Second, the positive emotional experience of special education teachers and improvement in their well-being needs attention. The well-being of special education teachers is relatively low. To improve the professional happiness of special education teachers, we can start from the following aspects: first, create a relaxed and warm education environment. Special education schools should start with creating a relaxed and warm working environment, give teachers certain rights of self-development, improve welfare, and formulate a fair and reasonable school management system. Second, we should pay attention to the physical and mental health of special education teachers and provide various support systems for them. Finally, the prestige and social status of special education teachers should be improved. Although in recent years, the state has paid more and more attention to special children and special education, but the social respect and attention to special education and special education teachers are insufficient. Therefore, governments at all levels can ensure the economic and social status of special education teachers by introducing relevant policies and regulations. At the same time, with the help of traditional media and network, we should vigorously publicize the significance of special education, create good public opinion for the development of special education, and improve the social reputation of special education teachers.

Third, the work engagement of special education teachers in their daily work may have mediating effect between EI and general well-being, which should be increased. By improving the working status and experience of teachers in their work practice, their well-being can be improved, and the positive impact of EI on the happiness of special education teachers can be improved. In order to stimulate the willingness and autonomy of special education teachers to actively participate and invest in work, it is necessary to establish an effective incentive mechanism. External material incentive and internal motivation are commonly used as individual incentive. Therefore, we can start from the following two aspects: on one hand, improve the welfare of special education teachers. Maslow’s hierarchy of needs theory also points out that material needs are the first needs of human beings. Only under the premise of satisfying such low-level needs can individuals pursue higher-level needs. On the other hand, we should stimulate the internal working motivation of special education teachers. Special education teachers’ recognition of their own professional development and the sense of happiness from professional development are important factors affecting their work engagement. Therefore, school administrators should provide teachers with more opportunities for further study and learning, and build an internal communication platform so that teachers can get more motivation for professional development.

### Limitations and Future Research

Eventually, after integrating the relationships among EI, work engagement, and well-being, the tests, comparisons, and analyses with structural equation modeling revealed the relevant influence paths and direct and indirect effects. However, this research has several limitations. First, although the sample was diverse on the location of special education teachers, the uneven development of the economy and special education may influence the report of teachers on well-being. Future research could select a more representative group of special education teachers to deeply analyze their characteristics in well-being. Second, would the difference between eastern and western culture enhance the distinct development and performance of EI and well-being? For teachers in China, who experience different interactive relationships, and renqing and mianzi in Chinese society affect their EI and well-being, which is worthy of further discussion.

## Data Availability Statement

The original contributions presented in the study are included in the article/supplementary material, further inquiries can be directed to the corresponding author/s.

## Ethics Statement

The studies involving human participants were reviewed and approved by the Ethics Committee of Beijing Normal University. The patients/participants provided their written informed consent to participate in this study.

## Author Contributions

WF designed the study, collected the data, and wrote the manuscript. WT analyzed the data and revised the manuscript. CW and SL wrote the manuscript. YW critically revised the manuscript for important intellectual content. All authors contributed to the article and approved the submitted version.

## Conflict of Interest

The authors declare that the research was conducted in the absence of any commercial or financial relationships that could be construed as a potential conflict of interest.

## Publisher’s Note

All claims expressed in this article are solely those of the authors and do not necessarily represent those of their affiliated organizations, or those of the publisher, the editors and the reviewers. Any product that may be evaluated in this article, or claim that may be made by its manufacturer, is not guaranteed or endorsed by the publisher.
